# Case-Based Learning in Clinical Biochemistry: Performance and Perception of MBBS Phase-I Students in Chhindwara Institute of Medical Sciences, Chhindwara, Madhya Pradesh, India

**DOI:** 10.7759/cureus.97044

**Published:** 2025-11-17

**Authors:** Kapil Raghuwanshi, Jyoti Nagwanshi, Bhupendra Kumar Jain, Sharad Manore, Sudhakar Petkar, Mahendra Gandhe

**Affiliations:** 1 Biochemistry, Chhindwara Institute of Medical Sciences, Chhindwara, IND; 2 Medicine, Chhindwara Institute of Medical Sciences, Chhindwara, IND; 3 Respiratory Medicine, Chhindwara Institute of Medical Sciences, Chhindwara, IND; 4 Psychiatry, Chhindwara Institute of Medical Sciences, Chhindwara, IND

**Keywords:** case based learning, : medical education, pathology and clinical biochemistry, student-centered learning, undergraduate medical curriculum

## Abstract

Background

Medical education is shifting from traditional lectures to interactive strategies that emphasize clinical application. Case-based learning (CBL) uses structured clinical scenarios to promote active learning and reasoning. This study evaluated the effect of CBL on performance and perception of undergraduate medical students in clinical biochemistry.

Methodology

A comparative cross-over quasi-experimental study was conducted among 100 MBBS Phase-I students (n=50 per group) at Chhindwara Institute of Medical Sciences. Two biochemistry topics were taught in a crossover design using traditional lectures (TL) and CBL. Student performance was assessed by two 50-mark tests, and feedback was collected through a 10-item questionnaire. Data are presented as mean ± SD or n (%). Paired t-tests were used for score comparisons, with p<0.05 considered significant.

Results

In Test 1, students exposed to CBL scored higher (37.9 ± 5.1) than those receiving TL (31.2 ± 4.3; t = 7.09, df = 98, p<0.00001). In Test 2, after crossover, CBL again outperformed TL (38.0 ± 4.7 vs 29.8 ± 3.6; t = 9.86, df = 98, p<0.00001). Feedback revealed that 88/100 (88%) of students reported improved application of biochemistry in clinical practice, 84/100 (84%) endorsed enhanced recall of clinically relevant points, and 90/100 (90%) supported introducing CBL in year one. Non-response rates were <10% across all items.

Conclusion

CBL significantly improved both academic performance and student satisfaction compared with TL. Its consistent benefit across two modules, supported by positive student perceptions, underscores its value for bridging theoretical knowledge with clinical application. Incorporating CBL into undergraduate curricula may strengthen conceptual learning and clinical preparedness.

## Introduction

Medical education has traditionally relied on instructor-led lectures and textbook-based learning, where students first acquire theoretical knowledge before applying it in clinical settings. Although this approach has remained widely used for decades, there is now a global shift toward more interactive and applied instructional methods. In India, the Department of Medical Education emphasizes the early clinical relevance of teaching strategies through real or simulated case-based learning [1].

Case-based learning (CBL) is one such approach. By using structured clinical scenarios, CBL encourages active participation, problem-solving, and the application of concepts. In contrast, traditional learning (TL) remains largely passive and theoretical. CBL fosters critical thinking and clinical reasoning by challenging students to integrate foundational science with real-life contexts [2].

This approach has particular importance in clinical biochemistry, where the ability to interpret laboratory results within a clinical framework is essential. Early adoption of CBL in MBBS Phase-I has the potential to improve conceptual understanding and clinical applicability, aligning well with the competency-based medical education (CBME) framework that promotes outcome-driven, integrated learning [1].

Despite its advantages, the adoption of CBL in Indian medical colleges is not without challenges, including the need for faculty training, time constraints, and limited resources. Nevertheless, existing reports suggest that students perceive CBL as more engaging and clinically meaningful than TL [2]. However, limited evidence is available from newly established Central Indian medical colleges regarding the early implementation and impact of CBL in clinical biochemistry. Against this background, the present study was undertaken to evaluate the effectiveness of CBL compared with TL in clinical biochemistry, using both academic performance and student perceptions as outcome measures.

## Materials and methods

Objectives

The objectives of this study were: (i) to compare mean test scores between case-based learning (CBL) and traditional learning (TL), (ii) to assess student perceptions on domains such as engagement and clinical applicability, and (iii) to evaluate the feasibility of incorporating CBL in MBBS Phase-I teaching. 

Study design and Participants

This comparative cross-over quasi-experimental study was conducted in the Department of Biochemistry at Chhindwara Institute of Medical Sciences, Madhya Pradesh, over two months (January-February 2025). Ethical approval was obtained from the Institutional Human Ethics Committee (Ref. No.: CIMS/Ethics Committee/2024/14599; dated 20 December 2024). A total of 100 MBBS Phase-I students from the 2024-25 batch were recruited using simple random sampling after providing written informed consent. The sample size (n=100) was determined by the total number of enrolled phase-I students during the 2024-25 academic year, ensuring full coverage. All students were eligible, and random allocation into two groups (n=50 each) was performed using a computer-generated random number list based on roll numbers.

Randomization and Instructional Procedure

**Figure 1:**
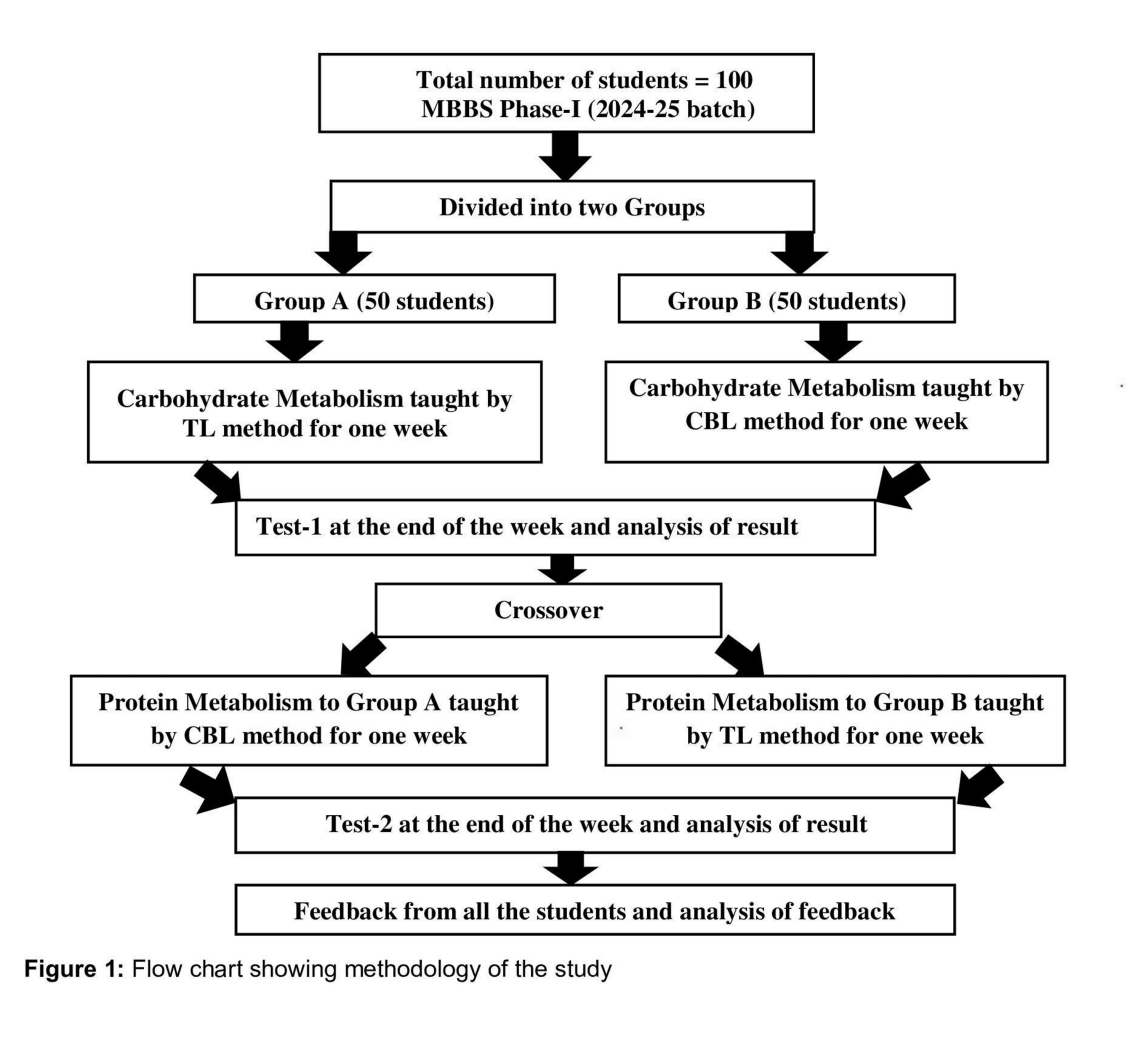
Flow chart showing methodology of the study.

Students were randomly allocated into two equal groups: Group A (n=50, 50%) and Group B (n=50, 50%). Two biochemistry topics of comparable difficulty-carbohydrate metabolism and protein metabolism-were selected. For standardized CBL, with the help of the medicine department, real clinical cases such as diabetes mellitus, diabetic ketoacidosis, lactose intolerance, protein energy malnutrition were selected, and some paper-based clinical cases, such as classical galactosemia, phenyl ketonuria, albinism, maple syrup urine disease were prepared. In the first phase, carbohydrate metabolism was taught to Group A by TL and to Group B by CBL. At the end of this phase, Test 1 was conducted. In the second phase, the groups were crossed over: Group A received CBL and Group B TL for protein metabolism, followed by Test 2. Both instructional sessions were delivered by the same faculty member to maintain uniformity across groups, and each session lasted approximately 60 minutes. Each CBL session was conducted in subgroups of eight to 10 students to facilitate discussion. In order to match learning objectives with CBME competencies such as problem-solving and clinical reasoning, we created quantifiable goals that mimic actual clinical and laboratory settings. The overall study design and crossover sequence are depicted in Figure 1.

Assessment of performance

Each test included 20 multiple-choice questions (1 mark each), two short-answer questions (5 marks each), and two case-based long-answer questions (10 marks each), totaling 50 marks. Scores were recorded as mean ± standard deviation (SD) for each group.

Student feedback questionnaire

A structured feedback questionnaire containing 10 items was distributed after the instructional sessions to evaluate perceptions regarding TL and CBL. The questionnaire underwent content validation by three senior faculty members. Internal consistency was measured, yielding a Cronbach’s alpha = 0.81, indicating good reliability. The items assessed domains such as engagement, clinical applicability, conceptual clarity, and teacher-student interaction. Responses were self-administered, anonymized, and recorded as n (%). Responses with missing data (<10%) were treated as missing without imputation. The questionnaire is presented in Table 1.

**Table 1:** Questionnaire for student feedback on CBL vs TL. CBL: case-based learning; TL: traditional lectures.

Serial Number	Questionnaire Item	Response Options
1	Was the CBL method the best to recall clinically relevant points?	Yes / No / Can’t say
2	Did the CBL method stimulate greater interest in the subject?	Yes / No / Can’t say
3	Did CBL cover a wide range of knowledge?	Yes / No / Can’t say
4	Did you understand the topic better using the CBL method?	Yes / No / Can’t say
5	Can the knowledge gained from CBL be applied in clinical practice?	Yes / No / Can’t say
6	Was conceptual learning possible through CBL?	Yes / No / Can’t say
7	Was the topic completed and covered well through CBL?	Yes / No / Can’t say
8	Was teacher–student interaction better during CBL?	Yes / No / Can’t say
9	Can CBL enable integration of Biochemistry with other medical subjects?	Yes / No / Can’t say
10	Should CBL be used to teach Biochemistry from the first year to promote clinical competence?	Yes / No / Can’t say

Statistical analysis

Continuous data were summarized as mean ± SD, and categorical data as n (%). Between-group comparisons of test scores were performed using independent t-tests, with results expressed as mean difference, t-statistic, degrees of freedom (df), and p-value. Categorical feedback responses were compared using chi-square (χ²) tests where appropriate. A two-tailed p-value <0.05 was considered statistically significant. Effect size (Cohen’s d) was computed to quantify the magnitude of between-group differences. Analyses were performed using Microsoft Excel (Microsoft Corporation, Redmond, USA).

## Results

Students demonstrated significantly improved academic performance following CBL compared with TL. In Test 1, Group A, which received CBL, achieved a mean score of 37.9 ± 5.1, while Group B, taught via TL, scored 31.2 ± 4.3. The difference was statistically significant (t=7.09, df=98, p<0.00001) (Table 2).

**Table 2:** Comparison of student performance in Test 1 following CBL and TL. Data are presented as mean ± SD. Between-group comparison performed using an independent t-test. Significance threshold: p<0.05. CBL: case-based learning; TL: traditional lectures.

Teaching Method	Group	N	Mean ± SD	Test statistic (df)	p-value
CBL	Group A	50	37.9 ± 5.1	t = 7.09, df = 98	<0.00001
TL	Group B	50	31.2 ± 4.3	—	—

**Figure 2:**
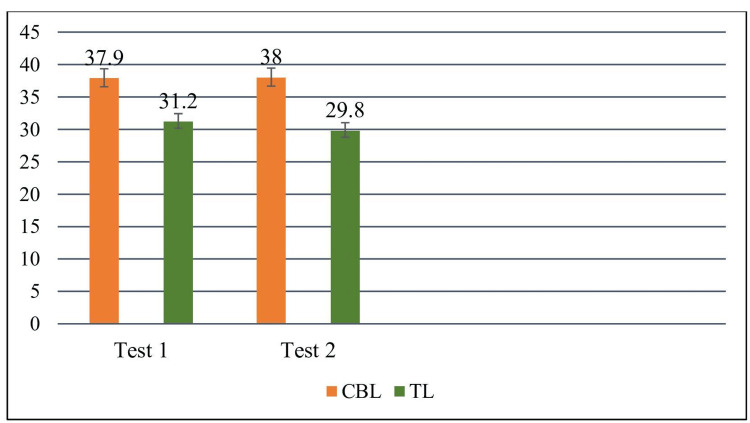
Comparison of marks obtained by students in test-1 and test-2.

In Test 2, after crossover, Group B scored 38.0 ± 4.7 under CBL, whereas Group A, now receiving TL, scored 29.8 ± 3.6. This difference was again significant (t=9.86, df=98, p<0.00001) (Table 3). The consistent six to eight mark increase under CBL across both modules supports its robust effect on knowledge acquisition. The crossover design strengthened internal validity by ensuring that all students were exposed to both methods. These comparisons are further depicted in Figure 2. Effect sizes were large (Cohen's d=1.41 for Test 1; d=1.88 for Test 2).

**Table 3:** Comparison of student performance in Test 2 following CBL and TL. Data are presented as mean ± SD. Between-group comparison performed using an independent t-test. Significance threshold: p<0.05. CBL: case-based learning; TL: traditional lectures.

Teaching Method	Group	N	Mean ± SD	Test statistic (df)	p-value
CBL	Group B	50	38.0 ± 4.7	t = 9.86, df = 98	<0.00001
TL	Group A	50	29.8 ± 3.6	—	—

Feedback analysis based on the structured 10-item questionnaire (Table 1) revealed strongly positive student perceptions of CBL. Out of 100 students, 88 (88%) reported that CBL improved their ability to apply biochemistry knowledge in clinical settings, while 84 (84%) agreed that it enhanced recall of clinically relevant points. In addition, 86 (86%) felt CBL covered a broader knowledge base, and 90 (90%) endorsed its integration into the first year of the MBBS curriculum. Interaction and engagement metrics were also favorable, with 82 (82%) of students reporting improved teacher-student interaction during CBL sessions, and 79 (79%) confirming that topics were thoroughly covered. Non-response rates remained below 10% across all items (Table 4). Facilitator observation checklists recorded higher student participation frequency (average five to six responses per session vs. two to three in TL), providing supporting evidence of increased faculty-student interaction during CBL.

**Table 4:** Student perceptions of case-based learning (CBL) in biochemistry. Data are presented as n (%). Chi-square (χ²) test was applied to categorical responses where appropriate. Significance threshold: p<0.05. CBL: case-based learning; TL: traditional lectures.

Serial Number	Student Perception Item	Yes n (%)	No n (%)	Can’t say n (%)	Non-Responder n (%)
1	CBL best for recalling clinically relevant points	84 (84%)	4 (4%)	5 (5%)	7 (7%)
2	CBL stimulated greater interest in subject	70 (70%)	12 (12%)	8 (8%)	10 (10%)
3	CBL covered a wide range of knowledge	86 (86%)	4 (4%)	3 (3%)	7 (7%)
4	Better understanding of topic through CBL	73 (73%)	13 (13%)	9 (9%)	5 (5%)
5	Knowledge from CBL applicable to clinical practice	88 (88%)	6 (6%)	4 (4%)	2 (2%)
6	Conceptual learning possible through CBL	81 (81%)	8 (8%)	6 (6%)	5 (5%)
7	Topic completed and covered well by CBL	79 (79%)	6 (6%)	7 (7%)	8 (8%)
8	Teacher–student interaction better during CBL	82 (82%)	9 (9%)	5 (5%)	4 (4%)
9	CBL enables integration with other medical subjects	85 (85%)	7 (7%)	2 (2%)	6 (6%)
10	CBL should be used from first year for clinical competence	90 (90%)	3 (3%)	3 (3%)	4 (4%)

Together, the performance outcomes and feedback results demonstrate both the academic effectiveness and acceptability of CBL in undergraduate biochemistry.

## Discussion

The present study shows that CBL significantly enhances both academic performance and student engagement when compared with TL. In Test 1, students taught by CBL achieved higher scores than those taught by TL (37.9 ± 5.1 vs. 31.2 ± 4.3; t = 7.09, df = 98, p < 0.00001). Similarly, in Test 2, after crossover, CBL again resulted in superior performance (38.0 ± 4.7 vs. 29.8 ± 3.6; t = 9.86, df = 98, p < 0.00001). These results are consistent across two modules, indicating that the benefit of CBL is not topic-dependent. Feedback data further support this finding, with 88% of respondents confirming that CBL facilitated clinical application of biochemistry, and 90% endorsing its inclusion from the first year.

Our findings align with existing literature. Tsekhmister [3] conducted a meta-analysis of 21 randomized controlled trials and reported consistent improvements in academic performance with CBL, reflected in a pooled standardized mean difference favoring this approach. The magnitude of gain in our study (~6-8 marks) is consistent with these results. In the Indian context, Ambike et al. [4] similarly reported that more than 85% of students believed CBL enhanced clinical knowledge and problem-solving ability, mirroring our feedback outcomes. Sanghani et al. [5] compared CBL with Interactive Didactic Lectures and observed greater post-test gains and higher motivation among CBL participants. Faculty in that study also rated CBL highly, echoing the systemic acceptability suggested by our results.

Large-scale investigations reinforce this evidence. Eissa et al. [6] demonstrated significantly higher post-test scores across two academic years involving more than 700 students each, while also noting that CBL improved teamwork and peer learning. Gasim et al. [7] reported a 92.4% satisfaction rate and over 65% strong agreement on the role of CBL in improving reasoning skills, comparable with our observation that over 80% endorsed CBL’s clinical relevance and integrative potential. Nair et al. [8] also documented significantly better performance with CBL (p < 0.0001) and widespread student preference, consistent with our 84% endorsement for improved recall of clinical information. Kaur et al. [9] found increased attendance and motivation under CBL, though without significant score differences. In contrast, our study showed both performance gains and strong student preference, suggesting that the small-group implementation here may have amplified the effect. Although small-group CBL requires substantial faculty efforts, scalable modifications(e.g., larger groups with breakout tasks, peer facilitation, digital case delivery) can extend feasibility to large classes with limited faculty. Collectively, these findings strengthen the external validity of our results across different populations and institutional contexts.

Strengths

This study incorporated a crossover design, ensuring that each student experienced both instructional methods and thereby reducing inter-group variability. Objective performance scores combined with subjective feedback provided a comprehensive evaluation of CBL’s impact. The use of a structured questionnaire enhanced the reliability of feedback, and the intervention aligned with competency-based medical education (CBME) goals of integrating clinical reasoning early in the curriculum. Importantly, the feasibility of implementing CBL in resource-limited settings highlights its scalability.

Limitations

This research measured short-term outcomes only; long-term retention and clinical application were not assessed. Being a single-center study with a homogenous student group, the generalizability of the results is limited. The sample size, while adequate, may be expanded in future multicenter studies. We did not control for potential confounders such as facilitator variability or prior student knowledge, which could have influenced outcomes. Feedback bias is also possible, as students may have responded favorably due to awareness of being observed (Hawthorne effect) or a tendency toward socially desirable responses.

Implications

The findings support broader curricular reforms to integrate CBL into preclinical teaching. Faculty development will be crucial for effective implementation, shifting the instructor’s role from lecturer to facilitator. CBL’s emphasis on clinical reasoning can bridge the gap between theory and practice earlier in training, thereby enhancing clinical readiness. Its potential for integration across disciplines also supports the CBME vision of competency-based, outcome-driven education. The strong student endorsement and significant performance gains observed in this study provide a foundation for policymakers and educators to consider wider adoption of CBL in undergraduate medical curricula. Future implementations should incorporate structured reflective exercises and self-assessment components to strengthen metacognitive skills, clinical reasoning, and lifelong learning attributes consistent with the CBME framework. Further studies assessing the long-term retention and application of CBL should use varied assessment methods, track students over longer periods, and incorporate more diverse and larger student groups.

## Conclusions

This study demonstrates that Case-Based Learning (CBL) significantly improves academic performance and student satisfaction in undergraduate clinical biochemistry compared with Traditional Lectures (TL). By fostering clinical reasoning and application of knowledge, CBL helps bridge the gap between theory and practice from the earliest stage of medical education. Given the strong performance gains and overwhelmingly positive student perceptions observed, integrating CBL into the undergraduate curriculum may enhance clinical preparedness and learner engagement. While TL retains value for structured knowledge delivery, CBL provides complementary benefits that support deeper understanding and long-term applicability. Broader adoption of CBL, supported by faculty training and institutional commitment, could strengthen competency-based medical education and improve the overall quality of medical training.
